# Antagonism to human BST-2/tetherin by Sendai virus glycoproteins

**DOI:** 10.1099/vir.0.051771-0

**Published:** 2013-06

**Authors:** Carole Bampi, Lara Rasga, Laurent Roux

**Affiliations:** Department of Microbiology and Molecular Medicine, Faculty of Medicine, University of Geneva, Geneva, Switzerland

## Abstract

Tetherin is an interferon-inducible factor that restricts viral particle production. We show here that Sendai virus (SeV) induces a drastic decrease in tetherin levels in infected HeLa cells. Using ectopic expression of tetherin in Madin–Darby canine kidney cells, we find that infectious SeV production is sensitive to restriction by tetherin, suggesting that SeV downregulates tetherin to counter this form of cellular restriction. By using radioactive tetherin in pulse–chase experiments, applying conditions that limit protein degradation, and by estimating tetherin mRNA levels, we find that tetherin degradation is the mechanism of downregulation. Suppression of the virus envelope proteins matrix, fusion (F) or haemagglutinin-neuraminidase protein (HN) during the course of infection demonstrates that F and HN, in concert, are responsible for tetherin degradation. The mechanism(s) by which these two viral glycoproteins participate in degrading tetherin remains to be determined.

## Introduction

Sendai virus (SeV) is a prototype of the family *Paramyxoviridae*, subfamily *Paramyxovirinae*, genus *Respirovirus*. It has been used, in the form of a non-defective (ND)/defective interfering (DI) particle mixed virus stock, as a potent interferon type I (IFN-β) inducer. ND SeV also has the ability to efficiently interdict IFN induction and IFN signalling. Although the complex interplay between SeV and the innate immune system has not yet been described in detail, it has been found to depend on the ability of the sensor RIG-I to detect infection and on viral V and C protein levels, which interfere with IFN induction and production of IFN-stimulated gene products (Burke *et al.*, 1998; Garcin *et al.*, 1999, 2001; Strahle *et al.*, 2006). Among these cellular products, tetherin (also called BST2, CD137 and HM1.24) has recently been found to harbour antiviral activity (Neil *et al.*, 2008). Tetherin is an unusual type II glycoprotein anchored at its N terminus and C terminus in cholesterol-rich membrane domains. The central part of the molecule contains a coiled-coil dimerization domain. Its mode of action is portrayed as physically restricting virion release, as well as promoting aggregation of viral particles (VPs), decreasing infectivity.

Tetherin has been shown effective against retroviruses [human immunodeficiency virus type 1 (HIV-1), HIV-2, simian immunodeficiency virus (SIV), equine infectious anemia virus, feline immunodeficiency virus] (Neil *et al.*, 2008; Le Tortorec & Neil, 2009; Jouvenet *et al.*, 2009), negative-strand RNA viruses (Ebola, Marburg, Nipah, Lassa, Machupo, vesicular stomatitis virus, influenza virus) (Radoshitzky *et al.*, 2010; Sakuma *et al.*, 2009; Weidner *et al.*, 2010; Mangeat *et al.*, 2012) and a member of the herpes virus family, human herpesvirus 8 (HHV-8), which is the agent causing Kaposi syndrome (Mansouri *et al.*, 2009). These findings have been made in the context of virus-like particle studies (Ebola, Marburg, Lassa/Machupo, Nipah, influenza virus) and during viral infections (influenza virus, HIV-1, SIV). In some cases, viral functions have been identified that counteract the restricting effect of tetherin by promoting tetherin degradation or by neutralizing its action; asVpu for HIV-1, nef for SIV, K5 for HHV-8 (Douglas *et al.*, 2009; Mitchell *et al.*, 2009; Jia *et al.*, 2009; Zhang *et al.*, 2009; Pardieu *et al.*, 2010) or viral envelope proteins (e.g. Env for HIV-2, NA for influenza virus; Le Tortorec & Neil, 2009; Yondola *et al.*, 2011).

This study analyses the interplay between tetherin and SeV. SeV particles contain two glycoproteins protruding from the envelope. The haemagglutinin-neuraminidase protein (HN) binds the cellular receptor and also contains a receptor cleaving activity. The fusion (F) protein fuses the viral envelope with the cellular membrane. A third envelope protein, the matrix (M) protein, carpets the inner layer of the envelope and bridges to the viral nucleocapsid (reviewed by Takimoto & Portner, 2004). To identify the role of each of these three proteins in the formation of VPs, HN, F and M were individually suppressed using small interfering RNA (siRNA), and HN and F were also suppressed in concert (Gosselin-Grenet *et al.*, 2010). As expected, M suppression led to a significant decrease in VP production. A similar decrease was observed upon F suppression (~10-fold), while suppression of HN was of no consequence. Surprisingly, the double suppression of F and HN led to the most significant decrease in VP production (~100-fold). These results partially reflected data obtained previously from experiments in which the cytoplasmic tails of F and HN were gradually truncated (Fouillot-Coriou & Roux, 2000). Removal of a TYTLE motif in the F cytoplasmic tail led to a significant loss of VP formation. In contrast, truncation or mutation of a SYWST motif in HN abolished HN incorporation into virions without decrease in the production of HN-less particles. Further characterizations of SeV particle formation showed that all SeV proteins were found in rafts. However, as raft association was equally observed upon M suppression and as mutated HN proteins not incorporated in VPs were equally found in rafts, the pertinence of rafts for SeV particle formation thus remains questionable (Gosselin-Grenet *et al.*, 2006).

The present study was initiated when we observed that SeV infections of HeLa cells resulted in a significant decrease of tetherin. Further suppression of tetherin using siRNA resulted in a 10-fold increase in infectious virus production. Ectopic expression of tetherin in Madin–Darby canine kidney (MDCK) cells equally reduced the number of physical and infectious particles produced, although tetherin level was again significantly reduced by SeV infection. These findings argued for a restrictive action of tetherin on SeV particle production and for a viral mechanism evolved to neutralize this restriction. Tetherin disappearance correlated with the extent of the infection and ^35^S-pulse–chase metabolic labelling demonstrated its faster turnover under infection conditions. Tetherin degradation was marginally affected by selective suppression of M, F or HN, individually. In contrast, suppression of F and HN together rescued tetherin levels, suggesting that the two surface glycoproteins act in concert to induce tetherin degradation.

## Results

### SeV infection reduces tetherin levels

**Fig. 1.**
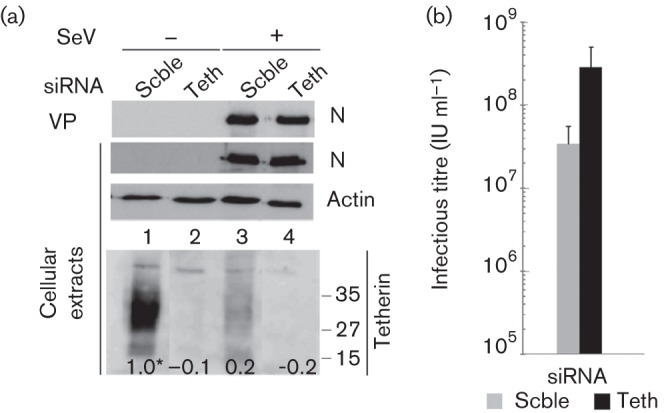
Effects of SeV infection on tetherin level and of tetherin suppression on VP production in HeLa cells. (a) HeLa cells were transfected with either scrambled (Scble) siRNA or a pool of tetherin-specific siRNAs (Teth) for 48 h before infection (m.o.i. = 20) with or without SeV (SeV +/−) for 24 h. Cell extracts were prepared and SeV N protein and actin levels were determined by Western blot analysis. Cell supernatants were collected and VPs were pelleted and quantified according to the N protein levels (VP). (b) Infectious particles in cell supernatants measured by flow cytometry and expressed as the number of infectious units (IU) ml^−1^. Bars represent deviation from the mean from three separate experiments. *, Partially saturated signal (see Methods).

HeLa cells constitutively express tetherin at detectable levels (Fig. 1a, cellular extracts, lane 1). The protein is seen as a diffuse band due to different glycosylation states. Tetherin expression is amenable to suppression by siRNA, after which all of the isoforms disappear (Fig. 1a, lane 2). When HeLa cells are infected with SeV at a high m.o.i. (m.o.i. = 20) and the tetherin level is probed at 36 h post-infection (p.i.), the signal is strongly reduced as well (Fig. 1a, lane 3). This level can be further decreased by siRNA suppression (Fig. 1a, lane 4). Notably, neither the intracellular level of infection nor the number of physical VPs in the supernatant, both monitored by the nucleocapsid protein (N), is affected by a further decrease of tetherin level (Fig. 1a, lanes 3 and 4, cellular extracts and VPs). In contrast, titration of the infectious units in the supernatant showed a 10-fold excess after further suppression of tetherin by siRNA (Fig. 1b). Thus, SeV infection is followed by a decrease of tetherin in the cells, and production of infectious SeV appears to be inversely proportional to tetherin levels.

### SeV production is sensitive to tetherin

**Fig. 2.**
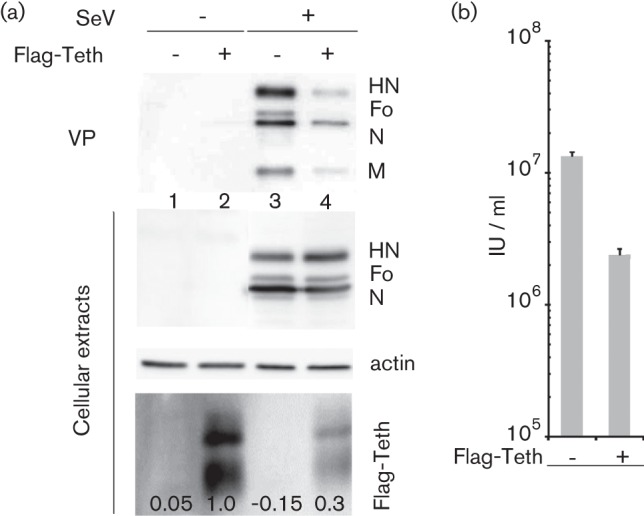
Effects of tetherin expression on virus particle production.**** (a) MDCK cells transduced with a lentiviral vector expressing Flag-Tetherin or with an empty vector (Flag-Teth +/−) were infected (m.o.i. = 10) with or without SeV (SeV+/−) for 24 h. Cell lysates and viral particles were analysed as in Fig. 1 by Western blots. (b) Titres of released infectious virus by flow cytometry. Mean of two separate experiments with deviation from the mean.

To confirm this observation, we used a MDCK cell line expressing a human Flag-tetherin (Flag-Teth; Mangeat *et al.*, 2012). This cell line was matched with a cell line derived from transduction with an empty vector such that direct comparisons of SeV infections in cells with and without tetherin were now possible. The production of physical particles, infectious units, and intracellular viral protein and tetherin levels were determined at 36 h p.i. Fig. 2(a) shows a deficit in physical virus particle production in the Flag-tetherin positive cells (SeV +, Flag-Teth +, VP lane 4) compared with the tetherin negative cells (Flag-Teth −, VP lane 3). A similar deficit is noted in the production of infectious units (Fig. 2b, Flag-Teth +). This deficit, however, is not observed at the level of intracellular viral proteins (Fig. 2a, cellular extracts, lanes 3 and 4). In addition, although SeV infection significantly reduces tetherin levels (compare lanes 2 and 4), some tetherin remains (Flag-Teth, lane 4) and this remaining amount is presumably responsible for a reduction in viral particle production. These data confirm that SeV is sensitive to the restrictive action of tetherin. This restriction apparently takes place at a late stage of viral multiplication, as there is no effect on the accumulation of viral proteins in the cells. They also show that the ability of SeV infection to reduce tetherin levels is not limited to HeLa cells. Tetherin is thought to tether viral particles to the cell membrane via dimerization of two tetherin molecules, one attached to a cellular membrane and the other to a viral envelope. Tetherin can also induce aggregation of VPs that nevertheless bud from cells (Douglas *et al.*, 2010; Evans *et al.*, 2010).

### Reduction in tetherin level is due to protein degradation

**Fig. 3.**
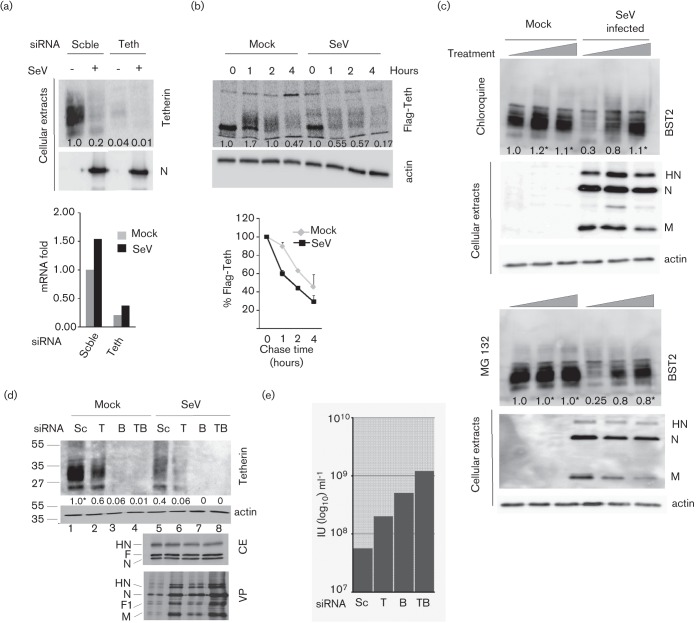
SeV infection induces tetherin degradation. (a) HeLa cells were transfected, infected (m.o.i. = 20) and analysed as in Fig. 1(a). Moreover, total RNA extracts were isolated and tetherin mRNA was determined by quantitative PCR (see Methods). (b) MDCK Flag-tetherin cells were mock-infected or infected with SeV (m.o.i. = 20). At 20 h p.i., cells were pulse radiolabelled for 15 min and chased for the indicated periods of time. Radiolabelled Flag-tetherin was immuno-precipitated, analysed by PAGE and revealed by autoradiography. Lower part: quantified values of tetherin were plotted. Bars represent mean sd from at least two independent experiments. (c) Mock-treated or SeV-infected (m.o.i. = 20) HeLa cells were incubated for 24 h in the presence of chloroquine (0, 25 and 50 µM) or the proteasome inhibitor MG132 (0, 0.4 and 0.8 µM). Tetherin levels were analysed by Western blot. Viral proteins and cellular actin were also monitored as controls. (d) HeLa cell samples were transfected with scrambled (Sc), anti-TIP47 (T), anti-tetherin (B) or anti-TIP47+anti-tetherin (TB) siRNAs 40 h prior to infection (m.o.i. = 20). Twenty-four hours p.i., cells and cell supernatants were collected. Cell extracts were analysed by Western blot for tetherin, actin and viral protein (CE) and infected cell supernatants for virus particles (VP). (e) Infectious titres in supernatants of infected cells in (d). *, Partially saturated signals (see Methods).

To gain insight into how tetherin levels were decreased, tetherin mRNA was measured by real-time PCR when tetherin protein was almost undetectable (Fig. 3a, upper panel, Scble). Compared to uninfected cells, no deficit in tetherin mRNA level could be observed following SeV infection (Fig. 3a, lower panel, Scble). If anything, a slight increase was noticed, possibly resulting from IFN induction following infection (see below). Tetherin siRNA treatment was used to verify that the mRNA detection methodology was adequate (Fig. 3a, Teth). In this case, a significant decrease in the tetherin mRNA level was recorded (Fig. 3a, lower panel, Teth). Next, tetherin protein synthesis and stability were monitored by metabolic pulse–chase labelling experiments. Tetherin protein was synthesized at a level comparable to that of uninfected cells (Fig. 3b, upper panel, time 0). However, following synthesis, tetherin loss appears faster in infected cells and is particularly evident at 1 h of chase. The lower panel of Fig. 3b illustrates that tetherin turnover is approximately two-fold faster after SeV infection. These results suggest that SeV infection decreases tetherin by enhancing its loss.

As tetherin is a trans-membrane protein expressed at the cell surface, its degradation is likely to involve internalization through the endocytic pathway to reach the lysosomes. Chloroquine treatment interferes with the efficiency of degradation through this pathway by preventing acidification of the endocytic compartment (Maxfield, 1982). As shown in Fig. 3(c), upper panel, chloroquine treatment of the infected cells led to tetherin recovery up to the level of uninfected cells, supporting the endocytic pathway as a route leading to degradation. In addition to degradation through the endocytic–lysosomal pathway, protein degradation can also take place via the proteasome. Treatment of the infected cells with MG132, a classical inhibitor of the proteasome (Lee & Goldberg, 1998), also resulted in increased levels of tetherin, arguing for the participation of the proteasome as well in tetherin degradation (Fig. 3c, lower panel).

The endocytic pathway is normally part of the tetherin recycling pathway, via the late endosome (LE) to the trans-Golgi network (TGN) where tetherin resides, a recycling that replenishes surface tetherin via the exocytic pathway (Kupzig *et al.*, 2003; Rollason *et al.*, 2007). After infection, the intense traffic of the viral HN and F proteins may interfere with this recycling, and direct tetherin to lysosomes. In an attempt to recreate conditions that would interfere with tetherin recycling, the 47 kDa tail-interacting protein (TIP47), known as an effector of LE to TGN trafficking (Díaz & Pfeffer, 1998; Ganley *et al.*, 2004), was suppressed by siRNA. Indeed, TIP47 suppression decreased tetherin levels in mock-infected and SeV-infected cells (Fig. 3d, upper panel, lanes T). In infected cells, this decrease was accompanied by a slight increase in the number of physical viral particles (lower panel, VP, compare lanes 5 and 6) and a five-fold increase in the number of infectious particles produced (Fig. 3e, sample T). Concomitant suppression of TIP47 and tetherin (lanes TB) produced the highest increase in SeV physical and infectious particles. In summary, these data suggest that tetherin loss is the result of faster degradation via trafficking pathways, plus proteasome activity.

### Time-course of tetherin degradation in SeV-infected cells

**Fig. 4.**
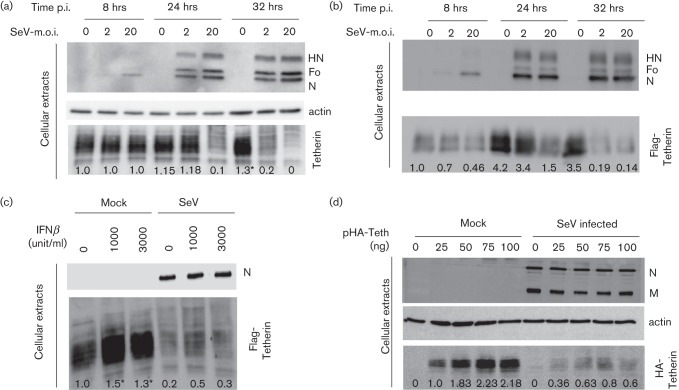
Tetherin degradation as a function of infection loads. (a) HeLa cells and (b) 293T cells expressing Flag-tetherin were mock-infected or infected with SeV (m.o.i. = 2 and 20) for 8, 24 and 32 h. Cell lysates were collected and protein levels were analysed by Western blot. (c) HeLa cells pre-treated during 16 h with increasing amounts of interferon β (0, 1000 or 3000 units ml^−1^) were mock-infected or infected with rSeV (m.o.i. = 20). At 24 h p.i., cell extracts and viral supernatants were analysed by Western blot. (d) HeLa cells were transfected with increasing amounts of a plasmid expressing HA-tagged tetherin (pHA-Teth), 0–100 ng) for 48 h and then infected with SeV (m.o.i. = 20). Cell extracts and viral supernatants were harvested at 24 h p.i. and the expression of the indicated proteins was analysed by Western blot. *, Partially saturated signals (see Methods).

Sendai virus infection generally has an eclipse phase of approximately 6–10 h. Maximum synthesis of viral proteins then takes place approximately 16–20 h p.i. and plateaus at approximately 24–30 h. Virus production is delayed by 4–6 h and stops when cells disintegrate. Fig. 4(a) illustrates these kinetics in HeLa cells. Eight hours p.i., intracellular viral proteins become detectable with differences depending on the m.o.i. These differences are still observed at 24 h p.i., while similar levels are reached by 32 h of infection. Tetherin levels remain stable at 8 h p.i., while at 24 h p.i., they have significantly decreased for an m.o.i. of 20. By 32 h, tetherin levels are decreased by more than six-fold (m.o.i. = 2) or undetectable (m.o.i. = 20). Interestingly, tetherin levels are increased at 32 h p.i. in mock-infected cells (m.o.i. = 0). This reproducible observation is attributed to suboptimal cell growth conditions (high confluency and/or low serum medium; see also Fig. 4b).

A similar profile is observed at 24 and 32 h p.i. in Flag-tetherin-expressing 293T cells (Fig. 4b). Notably, tetherin levels are low at 8 h p.i. and, as shown in Fig. 3(a), SeV infection does not direct tetherin degradation at this early time point. By 24 h, tetherin levels have increased in infected cells (as in mock-infected cells, shown here as SeV m.o.i. = 0), but now SeV infection promotes tetherin downregulation (especially for a m.o.i. of 20). At 32 h, under both infection conditions, tetherin levels become barely detectable. Increased tetherin levels could also result from induction by IFN, itself induced by SeV. That Flag-tetherin expression responds to IFN induction is shown in Fig. 4(c), where tetherin levels respond to IFN doses (Mock). In this case, SeV infection efficiently reduces even the excess of tetherin induced by IFN prior to infection. Finally, the ability of SeV infection to efficiently reduce tetherin amounts was tested, when regular 293T cells were transfected with increasing amounts of a HA-tagged tetherin-expressing plasmid (Fig. 4d).

These experiments show that tetherin degradation requires a threshold level of viral proteins. This level is reached at different times depending on the m.o.i. When this threshold level is reached, the effect of SeV infection is present regardless (constitutive expression in HeLa, ectopic expression in MDCK or 293T, induction by IFN). Taken together, these results are consistent with a direct effect of the viral proteins on tetherin degradation.

### Viral proteins involved in tetherin degradation

**Fig. 5.**
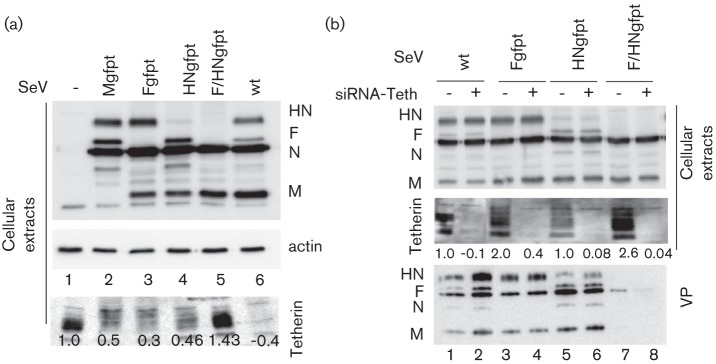
Effects of SeV protein suppression on tetherin fate. (a) M, F, HN or F and HN viral proteins were suppressed in si-gfpt-HeLa cells infected with wt, Mgfpt-, Fgfpt-, HNgfpt- or F/HNgfpt-rSeV (see Methods). Forty hours p.i., the cells and the supernatants were collected and analysed by Western blot to follow tetherin fate. (b) Tetherin was suppressed in si-gfpt-HeLa cells infected as in (a). Twenty-four hours p.i., cells and cell supernatants were collected. Cellular extracts and viral particles were analysed by Western blot.

In an attempt to clarify the mechanism of tetherin degradation, selective suppressions of viral proteins were performed using siRNA technology. Using this approach, M, F, HN or F+HN in concert were selectively suppressed upon infection with recombinant SeV (Mgfpt, Fgfpt, HNgfpt, F/HNgfpt) generated to carry a siRNA target in the 3′ untranslated region of the cognate mRNA(s) (see Methods for details and Gosselin-Grenet *et al.*, 2010). Fig. 5(a) (upper panel) illustrates the individual suppression of targeted proteins, which are basically undetectable. Of note, the general level of infection (the amount of N protein in each infection) remained equivalent. The lower panel shows the corresponding levels of tetherin found in the infected cells. Lane 6 (wt SeV infection) serves as the control for tetherin degradation, and lane 1 shows the level of tetherin in the uninfected cells. Individual suppression of M, F or HN appears to degrade tetherin less efficiently than in non-suppressed infection (compare lanes 2, 3 and 4 with lane 6). It is, however, only when both glycoproteins are suppressed that tetherin levels are restored to a level corresponding to uninfected cells. In fact, this level can even be higher, likely due to increased tetherin level induced by the infection. These data support the co-involvement of HN and F in tetherin degradation.

In a previous study (Gosselin-Grenet *et al.*, 2010), we analysed the basic requirements for SeV particle formation and production. We found that suppression of both HN and F led to an almost complete reduction of viral particle production. In light of the present results, it became of interest to analyse whether this could reflect the efficient tethering of neo-formed viral particles to the cell surface due to a lack of tetherin degradation. To this end, we suppressed tetherin by siRNA in conditions of HN and F co-suppression (Fig. 5b). Lane 8 (lower panel, VPs) shows that tetherin suppression by siRNA (middle panel, lane 8) does not restore production of virus particles lacking HN and F, confirming that suppression of HN and F per se is responsible for lack of viral particle production. This experiment also illustrates a partial restoration of tetherin levels after individual suppression of HN and F (lanes 3 and 5).

## Discussion

This study expands the list of viruses affected by tetherin to the *Paramyxoviridae*. SeV was first shown to promote degradation of constitutively expressed tetherin in HeLa cells (Fig. 1). SeV was then shown to similarly affect ectopically expressed tetherin, either in retrovirus transduced MDCK cells (Fig. 2), or in 293T cells after plasmid transfection (Fig. 4b). It equally affects tetherin induced by interferon treatment (Fig. 4c). Thus, tetherin degradation by SeV appears to be a general effect, independent of cell type or expression mode. Moreover, the results presented here possibly recapitulate the two modes of action of tetherin that have been described. In HeLa cells, tetherin suppression appears to mainly relieve virus particle aggregation, since the level of produced physical particle is not affected. In contrast, in MDCK cells, the infectious unit loss is paralleled by a decrease in physical particle consistent with a tethering of VPs to the cell membrane.

This degradation appears to follow the accumulation of viral proteins in the infected cells, and this supports a direct role of these proteins in the process. The fact that co-suppression of F and HN restores normal tetherin levels demonstrates this direct involvement. Fig. 5 clearly demonstrates that the two viral glycoproteins HN and F are responsible for tetherin degradation. Interestingly, co-suppression of HN and F appears to result in higher levels of tetherin than those achieved by the sum of the two individual suppressions, suggesting that HN and F act synergistically. This is the second time that a synergistic effect of the two surface proteins has been observed. HN suppression per se is of no consequence for the level of viral particle production. When co-suppressed with F, however, the absence of HN drastically reduces viral particle production, to a point that largely exceeds the effect achieved by F suppression alone (Gosselin-Grenet *et al.*, 2010). It thus appears that there is a particular step in virus particle production that requires the cooperation of the two surface glycoproteins. It is tempting to speculate that tetherin targets this particular step.

The involvement of viral glycoproteins in antagonizing tetherin has been reported for HIV-2 Env, EBOV GP (Le Tortorec & Neil, 2009) and influenza virus NA (Yondola *et al.*, 2011). In these cases, however, tetherin is not degraded but is likely downregulated from the cell surface. Tetherin degradation has rather been associated with ‘accessory’ viral proteins such as vpu for HIV-1 (Fritz *et al.*, 2012), nef for SIV (Jia *et al.*, 2009; Zhang *et al.*, 2009) and K5 for HHV-8 (Mansouri *et al.*, 2009; Pardieu *et al.*, 2010). Vpu’s mode of action is somewhat controversial depending on the cell type. It can interfere with normal recycling (via endocytosis and transfer to TGN) or send ubiquitinated tetherin to the lysosome or proteasome (discussed by Douglas *et al.*, 2010). SIV-nef enhances endocytosis, which likely results in addressing tetherin to lysosomes. HHV-8 K5 is an ubiquitin ligase that adds ubiquitin to a particular lysine (K18), thereby directing tetherin to the proteasome (Pardieu *et al.*, 2010).

In the case of SeV, tetherin degradation appears to follow the endocytic pathway as well as taking place in the proteasome. Whether this dual route is due to the two surface glycoproteins involved is an open question. Internalization of approximately 20 % of the cell-surface-expressed HN has been documented and HN has been identified in LEs (Gosselin-Grenet *et al.*, 2010). It is tempting, then, to postulate that HN interacts with tetherin and pulls it down the endocytic pathway, directing it to lysosomes. Attempts to show this interaction by co-immune precipitation, however, have failed (not shown). It remains that HNTL0, known not to be internalized due to mutation of the cytoplasmic tail SYWST motif (Gosselin-Grenet *et al.*, 2010), is found to have a marginal effect on tetherin degradation (Fig. S1, available in JGV Online). It is likely that this absence of internalization is shared by all HN mutants presented in Fig. S1. On the other hand, tetherin degradation appears not to be affected by F cytoplasmic tail truncation, arguing for the lack of participation of this domain in tethering degradation. Here again, attempts to demonstrate interactions of F and tetherin failed (not shown). Failure to show F-tetherin interaction and lack of apparent involvement of the F cytoplasmic domain in tetherin degradation may allude to interactions through the trans-membrane domains of the two partners, shown for vpu–tetherin interaction (Schubert *et al.*, 1996). Further experiments will be required to decipher the exact mechanism by which the two viral glycoproteins perform this task.

## Methods

### 

#### Virus and cells.

HeLa, MDCK and MK2 cells were grown at 37 °C under 5 % CO_2_ atmosphere in Dulbecco’s Modified Eagle Medium (DMEM; Gibco-Invitrogen) supplemented with 10 % fetal calf serum (FCS) and 1 % penicillin–streptomycin (PS; Sigma). Sendai virus (SeV) stocks were prepared in embryonated eggs and characterized as previously described (Roux & Holland, 1979). Recombinant SeV (rSeV) harbouring a 21 nt sequence tag (gfpt) from the green fluorescent protein (GFP) gene in the 3′ untranslated regions (UTR) of the M, HN, F, and both HN +F mRNAs have been described previously (Gosselin-Grenet *et al.*, 2010). A HeLa cell line constitutively expressing a siRNA against gfpt (si-gfpt-HeLa) was produced by transducing the cells with a lentivirus containing the specific siRNA sequence as described by Mottet-Osman *et al.* (2007). The MDCK cells expressing Flag-tetherin were a kind gift from Bastien Mangeat (Mangeat *et al.*, 2012).

#### Reagents and plasmids.

Antibodies include rabbit sera raised against SDS-denatured SeV HN, F, M or N proteins (α-HN_SDS_, α-F_SDS_, α-N_SDS_, α-N_SDS_; Mottet *et al.*, 1986). Monoclonal anti-HA epitope antibody (16b12) was purchased from Covance Research Products; monoclonal anti-actin antibody, clone C4, from Millipore; and anti-Flag antibody (F31565) from Sigma Aldrich. Rabbit polyclonal antibodies against tetherin made by K. Strebel were obtained from the NIH AIDS Research and Reference Reagent Program through Priscilla Turelli (EPFL, Lausanne, Switzerland). Peroxidase-conjugated secondary antibodies were purchased from Bio-Rad. The MG132 proteasome inhibitor and chloroquine were purchased from Sigma Aldrich. The expression plasmid pCDNA3.1 HA-tetherin was a kind gift from Bastien Mangeat (University of Geneva, Switzerland) and pCDNA3.1 was obtained from Invitrogen. IFN-β was obtained from Dominique Garcin (University of Geneva, Switzerland).

#### Plasmid and siRNA transfection.

HeLa cells in 35 mm-diameter dishes were transfected with 25 nM of a siRNA pool specific for tetherin RNA (siRNA ON-Target plus smart pool, # L-011817, Dharmacon) or scrambled siRNA (Qiagen) using Lipofectamine 2000 (Invitrogen) according to the manufacturer's instructions 48 h before SeV infection. Twenty-four hours before infection, HeLa cells were transfected with the expression plasmids pCDNA3.1 and pCDNA3.1 HA- tetherin with Lipofectamine 2000 according to the manufacturer's instruction.

#### Virus infections and radiolabelling.

Infections were performed at 33 °C. Virus stocks were adequately diluted (m.o.i. indicated in figure legends) in DMEM without FCS and laid over the cells for 1 h. At the end of the infection period, the infectious mix was removed and replaced with fresh DMEM supplemented with 2 % FCS. For radiolabelling, cells were incubated from 16 to 40 h p.i. with 40 µCi ml^−1^ ^35^S-methionine and ^35^S-cysteine (Pro-mix-[^35^S] *in vitro* cell labelling mix, Amersham Biosciences) in DMEM containing 1/10 the normal methionine and cysteine content plus 0.2 % FCS. Culture medium and cells were harvested at the times indicated in the figure legends and analysed as described below.

#### Cellular extracts and viral particle purification.

Transfected or infected cells were collected and disrupted in 300 µl Lysing Buffer II (150 mM NaCl, 1 % deoxycholate, 1 % Triton X-100, 0.1 % SDS, 10 mM Tris–HCl, pH 7.4) containing 1 % aprotinin and 20 mM AEBSF [4-(2-aminoethyl) benzenesulfonyl fluoride hydrochloride], as described previously (Mottet *et al.*, 1986). After 10 s of sonication (Branson Sonic Sonifer B-12, lowest speed), cell extracts were spun for 10 min at 12 000 r.p.m. in a microfuge. The supernatants were then processed for Western blot analysis or immune precipitation. VPs were isolated from the clarified cell supernatants by centrifugation through a 25 % glycerol cushion (Beckman SW55 rotor, 1 h, 43 000 r.p.m., 4 °C) and directly resuspended in SDS sample buffer.

#### Western blot analysis and autoradiography.

Total cellular extracts and VPs quantified by a Bradford assay were analysed by SDS-PAGE. After electrophoresis, the proteins were transferred using a semi-dry system (Bio-Rad) onto polyvinylidene difluoride membranes (Millipore). Blots were then incubated with specific antibodies, presented above, followed by HRP-conjugated secondary antibodies (Bio-Rad). Protein detection was performed using the enhanced chemiluminescence system (Amersham Biosciences) and signal was analysed by a FujiFilm Imager. The radiolabelled virus particle samples and the total immune-precipitate samples were analysed by SDS-PAGE and the gels, treated for enhanced fluorography (DMSO plus 5 % 2,5-diphenyloxazol-PPO), were exposed to Hyperfilm MP (Amersham Biosciences). Autoradiography scanned films as well as Western blot images were quantified using MutliGauge V3.0 (FujiFilm). Partially saturated signals detected by Multi Gauge V3.0 are indicated by an asterisk. Saturation of band signals takes place when the differences in band intensities between different samples are too high when one requires detection of weak signals. Although this leads to under estimation of protein levels, in all figures this under estimation strengthens our results rather than weakens them.

#### Viral supernatant titration.

Viral supernatant infectivity was measured according to Grigorov *et al.* (2011). LLC-MK2 cells were plated in 24-well plates (2×10^5^ cells per well) in the morning and infected in the afternoon with three-fold dilutions of the viral supernatants which were pre-treated for 30 min at 37 °C with 3 µg ml^−1^ acetylated trypsin. After a 20 h infection period at 33 °C, cells were detached with 150 mM NaCl and 5 mM EDTA and transferred to 96-well plates. Cells were then incubated for 30 min at 4 °C with an anti-F monoclonal antibody (F 38, obtained from Allen Portner, St Jude Children’s Research Hospital, Memphis, Tennessee, USA) diluted at 1/1000. Cells were then washed and incubated with a fluorescent secondary antibody (AlexaFluor 488 goat anti-mouse diluted at 1/500) for 30 min at 4 °C. Cells were then washed, fixed with 1 % paraformaldehyde and resuspended in PBS. The fluorescence level was analysed using a BD Biosciences Accuri C6 Flow cytometer. The titre was expressed as the number of infectious units per ml (IU ml^−1^) and calculated according to the formula: number of infected cells/volume of inoculation virus (ml).

#### Real-time RT-PCR.

Total RNA was extracted from cells with Trizol (Invitrogen) according to the manufacturer’s instructions. The integrity of the resulting RNA was checked on an agarose gel. This RNA served as templates for the synthesis of cDNA with random primers (Promega) at 42 °C using the M-MLV RNase (H-) point mutant reverse transcriptase (Promega). The cDNA was then quantified by real-time PCR on a TaqMan 7000 thermocycler (Applied Biosystems) with the following primers: tetherin (sense 5′-CTGCAACCACACTGTGATG-3′, antisense 5′-ACGCGTCCTGAAGCTTATG-3′) and probe O.FAM.BST2.1 (5′-FAM-TCCTTGGGCCTTCTCTGCATCCAG-BHQ-1-3′). The cycling conditions used were: 50 °C for 2 min, 95 °C for 10 min, 45 cycles of 95 °C for 15 s, and 60 °C for 1 min. GAPDH quantifications served as reference genes and allowed normalization for the starting amount of RNA.

#### Pulse–chase metabolic labelling of protein.

At 20 h p.i., MDCK Flag-tetherin cells were washed twice with DMEM lacking cysteine and methionine (DMEM -C/-M) (Sigma) then starved for 30 min at 37 °C in DMEM -C/-M. A 15 min pulse was performed in DMEM -C/-M with 300 µCi ml^−1^ ^35^S-methionine and ^35^S-cysteine (Pro-mix-[^35^S]). Cells were washed twice with DMEM enriched with 10 mM methionine, 10 mM cysteine and supplemented with 2 % FCS and 1 % PS. Cells were incubated for 0, 1, 2 or 4 h in the same medium and harvested. Cell extracts were lysed in Lysing buffer II containing 1 % aprotinin and 20 mM AEBSF, as described previously (Mottet *et al.*, 1986). The Flag-tetherin protein was recovered by immune precipitation. Total cellular extracts were first incubated with an anti-Flag antibody (F31565, Sigma Aldrich) overnight at 4 °C, then with a 50 % suspension of protein A-Sepharose (Roche) for 2 h at 4 °C.
